# Neutrophil Extracellular Traps in Viral Infections

**DOI:** 10.3390/pathogens14101018

**Published:** 2025-10-08

**Authors:** Jiajun Chen, Rong He, Jirong Luo, Shilu Yan, Wenbo Zhu, Shuangquan Liu

**Affiliations:** 1 Department of Clinical Laboratory Medicine, Institution of Microbiology and Infectious Diseases, The First Affiliated Hospital, Hengyang Medical School, University of South China, Hengyang 421001, China; 17872595140@163.com (J.C.); hr18007417750@163.com (R.H.); 17773300993@163.com (J.L.); 17873317537@163.com (S.Y.); 2 Hunan Province Clinical Research Center for Accurate Diagnosis and Treatment of High-Incidence Sexually Transmitted Diseases, The First Affiliated Hospital, Hengyang Medical School, University of South China, Hengyang 421001, China; 3 Clinical Medical Research Center, The First Affiliated Hospital, Hengyang Medical School, University of South China, Hengyang 421001, China

**Keywords:** immune evasions, immunologic injury, neutrophil, neutrophil extracellular traps, viral infections

## Abstract

Neutrophils are the most abundant immune cells in the human body. Neutrophil extracellular traps (NETs) have recently garnered significant attention as a novel, non-traditional mechanism for combating pathogenic microorganisms. Recent studies have shown that NETs play a crucial role in antiviral immunity, providing new perspectives on how neutrophils defend against viral invasion. Viruses not only induce NET formation through various mechanisms but have also developed multiple escape strategies targeting NETs. It is worth noting that NETs are a double-edged sword for the host: while they possess antiviral effects that inhibit viral spread and replication, their constituent components may also exacerbate tissue damage and play important pathological roles in the progression of certain viral infections. Therefore, a thorough understanding of the regulatory mechanisms and dynamic balance of NETs in viral infections is of critical importance. Additionally, since the components of NETs may vary depending on the stimulus, NET-related markers have the potential to serve as biomarkers for the severity and prognosis of viral diseases. This article provides a systematic review of the induction mechanisms, antiviral effects, viral escape strategies, and virus-induced NET-related immunopathological damage in viral infections, offering new insights for antiviral immunotherapy.

## 1. Introduction

Neutrophils are the first line of defense against pathogen invasion in the human body, excluding the skin, and are an important component of the innate immune defense. They are abundant in the circulatory system and contain abundant antimicrobial granules in their cytoplasm. They respond rapidly to pathogen invasion and infiltrate into inflammatory sites, playing a crucial role in pathogen clearance [1]. For a long time, it has been widely believed that neutrophils primarily combat pathogen invasion through phagocytosis, oxidative bursts, and degranulation [2]. However, this understanding expanded significantly with the introduction of a landmark study. In 2004, NETs were first identified and defined as a killing mechanism employed by neutrophils in response to specific stimuli, which are effective against multiple pathogenic bacteria. Over the subsequent two decades, this discovery has captured widespread attention among researchers, with research expanding to encompass other pathogens, fungi, protozoa, and, notably, viruses—with the first direct evidence of NETs targeting viruses emerging in 2012 [3]. Concurrently, research models have been extended to animals like fish and zebrafish, further confirming the cross-species conservation of this function. NETs are now established as a conserved immune mechanism spanning infection defense, immune regulation, and pathological injury, offering a novel perspective for understanding the immune functions of neutrophils [4,5,6,7].

The process of NET formation is referred to as NETosis, a form of programmed cell death distinct from apoptosis and necrosis. Within minutes of neutrophil stimulation and NETosis initiation, actin filaments begin to disassemble, halting cell movement and helping neutrophils anchor themselves at the site of inflammation. Subsequently, this is followed by the remodeling of vimentin filaments, endoplasmic reticulum vesicle formation, and chromatin decondensation. Once the nuclear membrane disintegrates, chromatin and cytoplasmic granules mix together to form intracellular NETs. Finally, the plasma membrane ruptures, releasing intracellular NETs into the extracellular space where they exert their immune-activating functions [8,9]. The essence of NETs is a fibrous network structure composed of a decondensed DNA scaffold and neutral granule-derived proteins attached to this scaffold, with a diameter of 15–17 nm. These derived proteins are highly diverse, including histones, citrullinated histones, serine proteases, myeloperoxidase, calmodulin, antibiotics, defensins, and cytoskeletal proteins, among others. However, the composition of NETs may vary depending on the stimulus neutrophils receive [7]. Once released into the extracellular space, NETs combat pathogen invasion by enveloping, capturing, and sustaining a pressure-driven microenvironment with high concentrations of antimicrobial proteins [4,10,11].

The classic pathway for NET release is known as suicidal NETosis, which relies on reactive oxygen species (ROS) generated by NADPH oxidase to activate the key enzyme—peptidyl arginine deiminase 4 (PAD4). PAD4 exhibits high ROS and calcium ion dependence, and its role in mediating the citrullination of histone arginine residues leads to a reduction in the positive charge of histones, thereby decreasing the electrostatic attraction between histones and negatively charged DNA and causing histone–DNA separation, nucleosome disassembly, and ultimately chromatin decondensation [12]. Patients with chronic granulomatous disease (CGD) have impaired NADPH oxidase complex 2, impairing or eliminating the ability of neutrophils to form NETs through ROS. These patients are extremely susceptible to fungal infections [13,14]. In some cases, the ROS produced by mitochondria are also sufficient to trigger NETosis [15,16]. In addition, there are non-canonical pathways for NET release that do not require neutrophil death, known as vital NETosis, which often activates PAD4 by inducing an increase in intracellular calcium concentration through the activation of calcium ion channels on the cell surface [17,18]. In this case, the extracellular release of NETs no longer depends on the rupture of the plasma membrane but rather on the transport of NETs to the extracellular space via nuclear budding and vesicles. At this point, neutrophils with intact cell membranes still retain a certain degree of phagocytic activity [19,20].

In previous studies, researchers generally deemed that NETs primarily play an immune role in bacterial and fungal infections. However, growing evidence suggests that they also play an important role in antiviral immunity. The antiviral mechanisms of NETs are more complex compared to their roles against other microorganisms. In addition to directly killing viral particles through antimicrobial proteins and restricting viral spread by enveloping them with sticky DNA structures, NETs can also exert antiviral immune effects indirectly by activating other immune cells, such as activating plasmacytoid dendritic cells (pDCs) [21] and lowering the activation threshold of T lymphocytes to enhance the body’s antiviral adaptive immune response [22]. However, it is worth noting that as toxic substances are released into the internal milieu, NETs can also impose a burden on the body and cause severe tissue damage while performing their antiviral functions [23,24]. Therefore, elucidating the relationship between NETs and viral infection is crucial for understanding and utilizing their immune functions. This article will review the role of NETs in antiviral immunity and related advances.

## 2. Materials and Methods

### 2.1. Search Strategy

We conducted literature searches following the PRISMA statement guidelines. The databases searched included PubMed, Web of Science, Embase, and other relevant databases, spanning the period from March 2004 (when NETs were first identified) to July 2025. Search terms included “neutrophil extracellular traps,” “NETs,” and “viral infection,” among others.

### 2.2. Inclusion Criteria

The inclusion criteria were as follows: (1) Basic science or clinical studies on the interaction between NETs and viruses. (2) Original studies (e.g., in vitro experiments, animal models, clinical studies) or high-quality reviews.

### 2.3. Exclusion Criteria

The exclusion criteria were as follows: (1) Duplicate publications. (2) Abstracts, meeting summaries, and similar publications that do not provide complete data. (3) Any literature with conflicts of interest.

### 2.4. Literature Screening and Quality Assessment

The titles, abstracts, and full texts of the retrieved literature were independently screened by two researchers, who cross-validated the results and evaluated the quality of the included studies. Finally, 110 articles that met the criteria were included.

## 3. The Mechanisms of Virus-Induced NET Formation

Recent studies have shown that the mechanisms by which viruses induce neutrophils to produce NETs exhibit high diversity, with different viral types and infection sites significantly influencing the response pathways of NETosis (Table 1). In most cases, neutrophils recognize viral nucleic acids or structural proteins through multiple receptors, thereby triggering NET formation. In certain cases, neutrophils are recruited to the microenvironment by various inflammatory mediators in the context of viral infection, indirectly inducing NET formation. Notably, platelets, as blood cells that become activated early in certain viral infections, can also bridge neutrophils and activate NET formation through multiple mechanisms.

Overall, these pathways do not exist in isolation or operate independently; rather, they coordinate NET formation through temporal coordination, signal crosstalk, or microenvironmental adaptation. Certain pathways may be rapidly activated in the early stages of infection to limit viral spread, while others remain persistently activated in the middle to late phases to counteract ongoing viral replication. Furthermore, the dominant activation patterns of these pathways adaptively adjust across different tissue microenvironments based on local signaling conditions, such as inflammatory mediators and hormone levels. This synergistic nature represents a key strategy for neutrophils in combating viral infections, offering multidimensional intervention targets for antiviral therapies targeting NETs. Specific mechanisms are elaborated in the subsequent sections.

### 3.1. Pattern Recognition Receptor-Mediated NET Generation

Neutrophil surface pattern recognition receptors (PRRs) play a key role in sensing viruses and activating subsequent signaling pathways.

The literature indicates that neutrophils can recognize human immunodeficiency virus (HIV) ssRNA via TLR7 and TLR8, subsequently activating the NADPH oxidase complex through the MyD88 pathway, inducing ROS production, and triggering the formation of NETs [3]. The nucleocapsid protein (N protein) and spike protein (S protein) of severe acute respiratory syndrome coronavirus 2 (SARS-CoV-2) can induce NET generation through multiple receptor-mediated pathways. Neutrophil surface receptors such as Dectin-1 and CLEC5A recognize linear glycosylated epitopes on the N protein and trimeric conformation-dependent glycosylated epitope on the S protein, recruiting the FcγRγ chain to activate the Syk kinase, which subsequently initiates downstream activation signals and induces neutrophils to produce non-ROS-dependent NETs [25]. Additionally, studies have shown that anti-S IgG1 antibodies produced by the host against the S protein can bind to Fcγ receptors on the surface of neutrophils with high affinity, activating the Syk-p38 MAPK signaling pathway through receptor cross-linking, thereby activating neutrophil oxidative burst and inducing the release of NETs [26]. Chikungunya virus (CHIKV) and respiratory syncytial virus (RSV) both induce NET formation via the PRRs-ROS axis, but their specific mechanisms differ significantly. When infecting mice and humans, CHIKV activates neutrophil NET generation by activating TLR7 [27]. RSV viral particles and their F protein can also induce NET release via a concentration gradient, but the core mechanism relies on the activation of the TLR4, ERK, and p38 MAPK signaling pathways [28]. Unlike CHIKV and RSV, Zika virus (ZIKV) non-structural protein 1 (NS1) mediates ROS-independent NET formation via TLR4 [29].

### 3.2. NET Formation Mediated by Functional Cell Surface Receptors

Different from the above mechanism that depends on PRR activation signaling pathways, multiple viruses can also trigger neutrophil NETosis through functional receptors on the cell surface.

Neutrophils can be activated by the HIV-1 envelope glycoprotein (Env) through the CCR5 and CXCR4 co-receptor pathway, leading to the activation of the phospholipase C-mediated calcium signaling pathway, resulting in a rapid increase in intracellular calcium concentration, which then activates PAD4, thereby inducing ROS-independent NETosis. In peripheral blood neutrophils, calcium ion signaling activated by the CCR5 and CXCR4 co-receptors dominates the early response, while ROS signaling activated by the TLR7/8 pathway dominates the late response [3,30]. It should be noted that aging can attenuate neutrophils’ responsiveness to calcium signaling, resulting in delayed NET release [30]. Nonetheless, neutrophils in the female reproductive tract have impaired responses to calcium signals, and the formation of HIV-1-induced NETs in this region mainly depends on the activation of late TLR8 signaling. This could explain why women are susceptible to HIV infection after high-risk sexual behavior, and this pathway is further impaired by aging [30]. Additionally, studies have shown that neutrophils in the cervix and endometrium release NETs more efficiently than peripheral blood neutrophils, which further highlights the important role of NETs in preventing HIV infection in the female reproductive tract [31]. However, this appears to be partially inconsistent with the notion of calcium signaling defects in neutrophils in the female reproductive tract, which requires further investigation.

Additionally, hantavirus (HTNV)-induced NETs depend on the functional cell surface receptor β2 integrin (CD18), whose heterodimeric subunits CR3 (CD11b/CD18) and CR4 (CD11c/CD18) jointly mediate the viral infection process in neutrophils. Studies have shown that neutrophils in β2 integrin gene knockout mice completely lose their ability to respond to HTNV stimulation, and thus they are unable to induce NET formation by activating NADPH oxidase to produce ROS [32].

### 3.3. NET Formation Mediated by Indirect Stimulation and Inflammatory Environment

In addition to the direct activation of neutrophils by viruses, neutrophils can also produce NETs in response to various indirect stimuli during viral infection.

Hepatitis B virus (HBV) infection leads to the downregulation of MHC-I molecule expression levels in hepatocytes, rendering the inhibitory receptors on NK cell surfaces unable to recognize their ligands and thus becoming disabled. This process will activate NK cells, which release perforin and granzyme B. Perforin forms small pores in the hepatocyte membrane, facilitating the entry of granzyme B into the cell. Once granzyme B enters hepatocytes, it will induce the activation of caspase-8 and the cleavage of GSDMD, leading to hepatocyte pyroptosis. The integrity of the plasma membrane is disrupted during pyroptosis, resulting in the release of High Mobility Group Box 1 (HMGB1) into the extracellular environment. HMGB1 will activate TLR4 signaling, which in turn triggers the release of NETs by neutrophils [33]. At the same time, HBV infection can upregulate the expression of S100A9 in hepatocellular carcinoma cells. Upon secretion, this protein can activate NADPH oxidase through TLR4/RAGE receptors, thereby promoting the formation of NETs [34]. In addition, in patients with HBV-induced fulminant viral hepatitis (FVH), the expression of Neutrophil-specific fibrinogen-like protein 2 (FGL2) and an ion channel protein called mucolipoprotein 3 (MCOLN3) is upregulated in neutrophils. FGL2 can directly interact with MCOLN3, regulating calcium ion influx and initiating autophagy, thereby leading to the formation of NETs [35]. It is worth noting that the multiple mechanisms by which HBV induces NET formation may result in heterogeneous NET composition, which depends on the course of viral hepatitis. Hepatitis patients with elevated levels of NET markers, such as citrullinated histone (Cit H3) and HMGB1, may indicate HBV infection, while concurrent increases in autophagy-related proteins may be associated with the progression of fulminant hepatitis. This suggests that NETs have potential as biomarkers for liver disease stratification [33,34,35]. Infection with parvovirus B19 (B19V) induces the production of B19V-VP1u IgG antibodies in the host. These antibodies can induce ROS-dependent NETosis by activating the cAMP/PKA signaling pathway in neutrophils, but this process does not depend on PAD4, suggesting that other non-classical NETosis pathways may exist in the context of B19V infection [36].

Also, the inflammatory environment formed after viral infection is often enriched with high concentrations of inflammatory factors, such as IL-1β, IL-6, IL-8, and TNF-α, among others [37,38]. On the one hand, neutrophils are recruited to the site of infection by these inflammatory mediators in the host body. On the other hand, these inflammatory mediators enhance their oxidative burst capacity, indirectly inducing the ability to generate NETs. Taking varicella-zoster virus (VZV) infection as an example, VZV activates a strong type I interferon (IFN-α/β) response in the host after infection, leading to the release of large amounts of inflammatory factors and chemokines, which induce the explosive release of NETs [39]. Similarly, rhinovirus (RV) infection specifically stimulates epithelial cells to release IL-33, which is a unique alarm molecule produced by epithelial cells. On the one hand, IL-33 promotes neutrophil chemotaxis; on the other hand, it binds to ST2 on the surface of neutrophils, activating the PLCγ2–calcium signaling pathway and inducing the release of NETs. In patients with asthma complicated by RV infection, this activation pattern is even more pronounced, ultimately leading to the exacerbation of asthma symptoms [40]. In studies of influenza A virus (IAV), alveolar macrophages recognize viral RNA through Z-DNA-binding protein 1 (ZBP1), triggering RIP3-dependent necroptosis and the release of IL-1α/β, which promotes neutrophil chemotaxis, activates their IL-1R receptors, and subsequently promotes NET formation [41].

### 3.4. NET Formation Mediated by the Synergistic Effect of Neutrophils and Platelets

In the case of viral infection, platelets are usually in a highly activated state [42,43]. Research indicates that activated platelets play an important role in the generation of NETs [44].

H1N1 is a subtype of IAV. In mice infected with the H1N1 influenza virus, large numbers of platelets and neutrophils were observed to accumulate in the pulmonary blood vessels [24]. Research shows that H1N1 infection induces the expression of tissue factor (TF) in lung cells, activating the plasminogen activator. The plasminogen activator activates PAR4 by cleaving proteases on the surface of platelets, promoting platelet aggregation and activation. Activated platelets release chemokines and microparticles, promoting the recruitment of neutrophils. Additionally, through the binding of surface P-selectin to neutrophil surface P-selectin glycoprotein ligand 1 (PSGL-1), they trigger the activation of neutrophil surface integrin CD18, enhancing their binding and activating the NF-κB and MAPK signaling pathways within neutrophils, thereby inducing the release of NETs [24]. Other studies have shown that in dengue virus (DV) infection, the NS1 of DV can directly activate platelets, promoting platelet degranulation through the TLR4 signaling pathway and releasing P-selectin and von Willebrand factor (VWF), enhancing platelet–neutrophil interactions and inducing neutrophils to release ROS-independent NETs. Inactivated NS1 co-incubated with platelets failed to induce this effect [45]. Additionally, after DV activates platelets, they release exosomes (EXOs) and microvesicles (MVs), which induce NET release through the CLEC5A and TLR2 signaling pathways, respectively [46].

The above mechanisms indicate that platelets not only play a key role in coagulation disorders associated with viral infections, but they also serve as an important link between viral infections and NET-related immune responses. The excessive formation of NETs not only exacerbates inflammatory responses, but it also may accelerate multi-organ damage and coagulation disorders in patients with dengue hemorrhagic fever and COVID-19 [47,48]. Therefore, in the study of virus-related diseases, it is crucial to explore the balance between platelet activation and NET release.

**Table 1:** Molecules and mechanisms involved in virus-induced NET formation.

Mechanism	Virus	Molecule	Specific Results	ROS Dependencies	Documents
Direct activation of PRRs	HIV	ssRNA	ssRNA acts as a PAMP and is recognized by the host TLR7/TLR8, activating the MyD88-dependent signaling pathway.	Yes	[3]
	SARS-CoV-2	Nucleocapsid protein	CLR recognizes N-protein glycosylation structures, recruits Syk through the FcγRγ chain, and activates downstream pathways.	No	[25]
		Spike protein	CLR recognizes the glycosylation structure of the S protein, recruits Syk through the FcγRγ chain, and activates downstream pathways.	No	[25]
			The Fc region of anti-S protein IgG1 antibodies cross-links Fcγ receptors, directly activating the Syk–p38 MAPK signaling axis.	Yes	[26]
	CHIKV	ssRNA	ssRNA acts as a PAMP, recognized by TLR7 in neutrophil endosomes, activating downstream pathways.	Yes	[27]
	RSV	F protein	The F protein directly binds to the TLR-4/MD-2 complex, activating downstream pathways.	Yes	[28]
	ZIKV	NS1	NS1 is recognized by host TLR4, activating downstream pathways.	No	[29]
Activation of functional receptors	HIV	Env	Env activates the phospholipase C-calcium signaling pathway through the co-receptors CCR5 and CXCR4.	No	[30]
	HTNV	Structural protein	HTNV binds to the heterodimeric receptors CR3 and CR4 of β2 integrin, activating Src family kinases and triggering downstream signaling pathways.	Yes	[32]
Indirect stimulation by other inflammatory molecules	HBV	HMGB1	HBV activates NK cells to release perforin and granzyme B, inducing hepatocyte pyroptosis and HMGB1 release, HMGB1 binds to neutrophil TLR4, triggering downstream signaling.	-	[33]
		S100A9	S100A9 binds to TLR4 and RAGE, activating Src kinase and downstream signaling.	Yes	[34]
		FGL2	HBV infection upregulates FGL2 expression, which binds MCOLN3 to mediate calcium influx.	No	[35]
	B19V	VP1	The unique N-terminal region of VP1 (capsid protein VP1u) induces anti-VP1u IgG production, then activates the cAMP/PKA pathway.	Yes	[36]
	VZV	Inflammatory factors	VZV activates a strong type I interferon response, recruiting neutrophils and activating NETosis through multiple inflammatory factors.	-	[39]
	RV	IL-33	RV induces IL-33 release from airway epithelial cells, which activates neutrophil ST2 and triggers downstream signaling.	-	[40]
	IAV	IL-1α/β	Alveolar macrophages detect viral RNA via ZBP1, inducing RIP3-dependent necroptosis and IL-1α/β release, which activates neutrophil IL-1R and downstream signaling.	-	[41]
Synergistic action of platelets	IAV(H1N1)	TF	H1N1 induces tissue factor expression in lung tissue, activates thrombin, cleaves platelet PAR4, promotes platelet activation, binds neutrophils via PSGL-1, activates neutrophil CD18, and triggers downstream signaling.	-	[24]
	DV	NS1	NS1 activates platelets via TLR4 signaling, enhances platelet–neutrophil adhesion, and triggers neutrophil downstream signaling.	No	[45]
		-	DV binds platelet CLEC2, activating Syk kinase and triggering degranulation with extracellular vesicle (EV) release. EVs then bind neutrophil CLEC5A and TLR2.	-	[46]

HIV, human immunodeficiency virus. SARS-CoV-2, severe acute respiratory syndrome coronavirus 2. CHIKV, chikungunya virus. RSV, respiratory syncytial virus. ZIKV, zika virus. HTNV, hantavirus. HBV, hepatitis B virus. B19V, parvovirus B19. VZV, varicella-zoster virus. RV, rhinovirus. IAV, influenza A virus. H1N1, influenza A virus subtype H1N1. DV, dengue virus. ssRNA, single-stranded RNA. NS1, non-structural protein 1. Env, envelope glycoprotein. FGL2, fibrinogen-like protein 2. VP1, viral capsid protein 1. TF, tissue factor.

## 4. Antiviral Mechanisms of NETs

**Figure 1:**
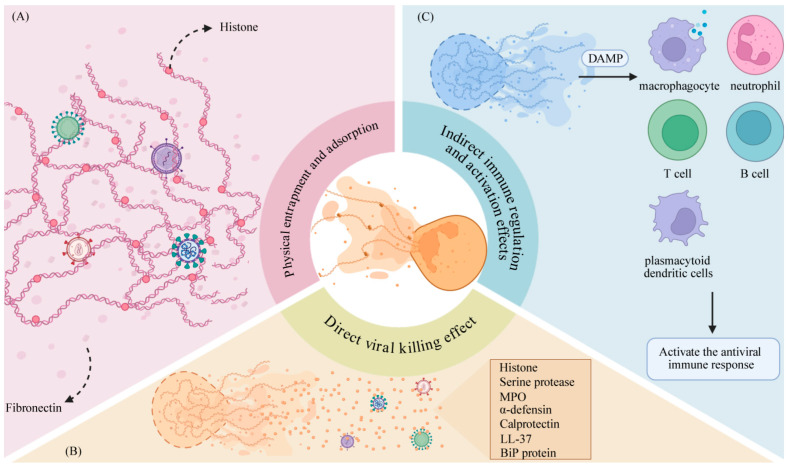
The antiviral mechanisms of NETs. (**A**): NETs are anchored in the interstitium by fibronectin and capture viruses through their sticky DNA scaffold and positively charged histones. (**B**): NETs directly combat viral pathogens through an arsenal of neutrophil-derived proteins with virucidal or inhibitory functions. (**C**): NETs contribute to antiviral immunity by releasing DAMPs that activate and recruit other immune cells, thereby initiating a broader immune cascade.

NETs represent a crucial mechanism by which neutrophils exert their antiviral functions, employing multiple synergistic pathways to counter viral invasion (Figure 1). Central to this process is the entrapment of viruses within a concentrated microenvironment enriched in cytotoxic proteins, which suppress viral replication and dissemination or directly compromise viral structural integrity. Additionally, NETs can activate other immune cells, triggering a cascade amplification of antiviral immunity. These mechanisms not only highlight the critical role of neutrophils in combating viral invasion but also reveal the dynamic regulatory function of NETs as immune signaling hubs, offering a novel perspective on virus–host interactions.

### 4.1. Physical Entrapment and Adsorptionlatelets

NETs can mechanically bind and capture viruses, preventing their spread [31]. The DNA scaffold of NETs is highly viscous, and its decondensation process significantly increases the exposed surface area in the extracellular environment [49]. Concurrently, the highly expressed fibronectin in the extracellular matrix contains DNA-binding sites, which facilitate the anchoring of NETs and help them resist the effects of blood flow shear stress, thereby enhancing the virus-capturing capacity of NETs [29,50,51]. Additionally, histones embedded in the NET structure are rich in positively charged amino acids, which can adsorb negatively charged viral particles and envelopes through electrostatic interactions [3,52].

### 4.2. Direct Viral Killing Effect

Multiple neutrophil-derived proteins that are attached to NETs can directly kill viruses, inhibit viral replication, or block viral transmission. According to research reports, histones H1 and H2A in NETs, along with myeloperoxidase (MPO) and α-defensin, can inhibit HIV-1 transcription and disrupt the integrity of viral envelopes [3]. In RSV infection, MPO inhibits the in vitro replication of RSV isolates, and this effect can be reversed by MPO inhibitors [53]. Additionally, serine proteases and bactericidal/permeability-increasing protein (BPI) in NETs can recognize, bind, and cleave critical sites of the RSV F protein, thereby blocking RSV fusion with host cell membranes and intercellular spread [54,55].

### 4.3. Indirect Immune Regulation and Activation Effects

Neutrophil-derived proteins can also exert antiviral effects indirectly by stimulating and activating the antiviral responses of other immune cells. The literature indicates that histones and HMGB1, as DAMPs, can activate other immune cells to release pro-inflammatory cytokines to trigger immune responses [56,57] or activate pDCs through TLRs to initiate the type I IFN antiviral response, thereby activating a cascade amplification of antiviral immunity [21]. Additionally, NETs can enhance the speed and extent of the body’s antiviral immune response by lowering the activation threshold of T lymphocytes [22].

## 5. The Strategies for Viral Escape from NET-Mediated Killing

NETs are important immune defense weapons of neutrophils, but viruses have also evolved to have powerful mechanisms to evade host immune responses. They have developed various strategies to evade the cytotoxic effects of NETs, thereby creating favorable conditions for their survival, replication, and spread within the host (Table 2). The main escape strategies include inhibiting neutrophil activation, blocking the release pathways of NETs, interfering with neutrophil metabolism, and disrupting the structural framework of formed NETs. Notably, different viral types exhibit significant differences in their escape strategies. This dynamic interplay between viruses and hosts not only reveals the critical role of NETs in antiviral immunity but also, by elucidating these viral escape mechanisms, provides some new insights into the development of antiviral therapies targeting NETosis regulation.

### 5.1. The Role of Immunosuppressive Factors

IL-10 is a classic immunosuppressive cytokine that downregulates the expression of activation signals on the surface of neutrophils, suppresses their oxidative burst capacity, and thereby effectively attenuates neutrophil activation [58]. In studies of HIV-1 infection, it has been demonstrated that the virus binds to DC-SIGN (CD209, a dendritic cell-specific C-type lectin expressed on the surface of dendritic cells) via its envelope protein gp120 and then spreads along the infectious synapses formed between CD209^+^ dendritic cells and CD4^+^ T cells, leading to efficient infection of CD4^+^ T cells by HIV-1. Notably, HIV-1 carrying envelope glycoproteins can induce dendritic cells to secrete CD209-dependent IL-10, thereby inhibiting neutrophil activation and NET formation [3]. In patients with preeclampsia (PE), the placenta typically exhibits a high expression level of Syncytiotrophoblast-derived microparticles (STBMs) and IL-8, which will activate neutrophils and induce the formation of NETs. However, in patients with HIV and PE, placental NET levels are significantly reduced, and we speculate that this phenomenon may be due to the HIV-mediated immune suppression of NETs [59]. In addition, previous studies have shown that the genomes of some large DNA viruses can encode molecules similar to IL-10, including common Epstein–Barr virus (EBV) and human cytomegalovirus (HCMV). These virus-derived IL-10 analogs can also inhibit the function of neutrophils and even cause cell death, thereby enabling the viruses to escape from NETs [60,61].

### 5.2. Pathway Interference and Metabolic Hijacking

Oxidative stress plays a key role in the activation of ROS-dependent NET formation. However, certain viruses can prevent NET formation by interfering with the neutrophil oxidative stress pathway. Studies have found that the core protein HBcAg/HBeAg of HBV can inhibit the phosphorylation of ERK1/2 and p38MAPK, thereby blocking the oxidative burst of neutrophils and reducing ROS-dependent NET formation [62]. Acute-on-chronic liver failure (ACLF) caused by HBV infection is a syndrome characterized by a systemic inflammatory response and impaired antibacterial immune function, posing a serious threat to patients’ lives. Studies have shown that in ACLF patients, neutrophil-derived NET formation significantly increases upon stimulation, but NET-associated DAMPs such as HMGB1 and HSP70 do not show marked elevation. Furthermore, this dysfunctional increase in NETs is associated with poor patient outcomes, suggesting that neutrophil dysfunction occurs in ACLF patients. Nonetheless, the underlying mechanisms require further investigation [63].

Additionally, the NETosis process is accompanied by a significant depletion of neutrophil bioenergetics. Studies have reported that glucose consumption increases over time during NET formation, concurrent with a rise in the expression of the glucose transporter GLUT1 [64]. However, compared to neutrophils treated with PMA alone, cells incubated with dengue virus serotype 2 (DENV2) exhibited significantly reduced GLUT1 expression and glucose uptake capacity, along with an impaired ability to release NETs. This phenomenon suggests that DENV2 employs a strategy of hijacking neutrophil energy metabolism to evade the immune response [65].

### 5.3. Directly Disrupting the Structure of NETs

Pathogens such as bacteria or fungi typically directly disrupt the established NET structure by secreting extracellular nucleases (DNase I), thereby evading the killing action of neutrophils [66,67]. Although there are currently no reports of viruses directly secreting DNase I, viruses can indirectly achieve the goal of degrading NETs by inducing the host to upregulate its own DNase I levels. HBV-induced cirrhosis leads to a hypoxic environment in hepatocytes, which induces the expression of the hypoxia-inducible factor HIF-1α. HIF-1α can directly bind to the hypoxia response element on the DNASE1 promoter, thereby upregulating the host’s DNase I levels [68]. Additionally, some viruses encode and express proteins with nuclease activity. The non-structural protein nsp14 of SARS-CoV-2 possesses 3′-5′ exonuclease activity [69], while herpes simplex virus (HSV) encodes Vhs [70], IAV encodes PA-X [71], Kaposi’s sarcoma-associated herpesvirus (KSHV) encodes SOX [72], and SARS-CoV-2 encodes nsp15 [73], all possessing nucleic acid endonuclease activity. These nucleases may play a role in disrupting the structural framework of NETs.

**Table 2:** Molecules and mechanisms of viral escape from NETs.

Immune Escape Mechanism	Virus	Molecule	Specific Results	Documents
Induce or express immunosuppressive factors	HIV	gp120	Binds to CD209 on dendritic cells, inducing the production of IL-10.	[3]
	EBV	BCRF1	Simulates IL-10 activity.	[60]
	HCMV	cmvIL-10	Simulates IL-10 activity.	[61]
Block signaling pathways	HBV	HBcAg/HBeAg	Inhibits the phosphorylation of ERK1/2 and p38MAPK, suppressing neutrophil oxidative burst.	[62]
Suppress metabolism	DENV2	-	Reduces GLUT1 expression and glucose uptake in neutrophils.	[64,65]
Induce or express nucleases	HBV	-	Cirrhosis causes hypoxia in liver cells, inducing the expression of HIF-1α in host cells and increasing the level of DNase I in the host.	[68]
	SARS-CoV-2	nsp14, nsp15	nsp14 possesses 3′-5′ exonuclease activity, while nsp15 possesses endonuclease activity.	[69,73]
	HSV	Vhs	Vhs has nucleic acid endonuclease activity.	[70]
	IAV	PA-X	PA-X possesses nucleic acid endonuclease activity.	[71]
	KSHV	SOX	SOX has nucleic acid endonuclease activity.	[72]

HIV, human immunodeficiency virus. EBV, Epstein–Barr virus. HCMV, human cytomegalovirus. DENV2, dengue virus serotype 2. HBV, hepatitis B virus. SARS-CoV-2, severe acute respiratory syndrome coronavirus 2. HSV, herpes simplex virus. IAV, influenza A virus. KSHV, Kaposi’s sarcoma-associated herpesvirus. gp120, glycoprotein 120. BCRF1, BamHI C fragment region 1. cmvIL-10, cytomegalovirus interleukin-10 homolog. HBcAg, hepatitis B core antigen. HBeAg, hepatitis B e antigen. nsp14, non-structural protein 14. nsp15, non-structural protein 15. Vhs, virion host shutoff protein. PA-X, polymerase acidic-X protein. SOX, shutoff and exonuclease. -, unknown.

## 6. NET-Mediated Host Immunopathological Mechanisms

**Figure 2:**
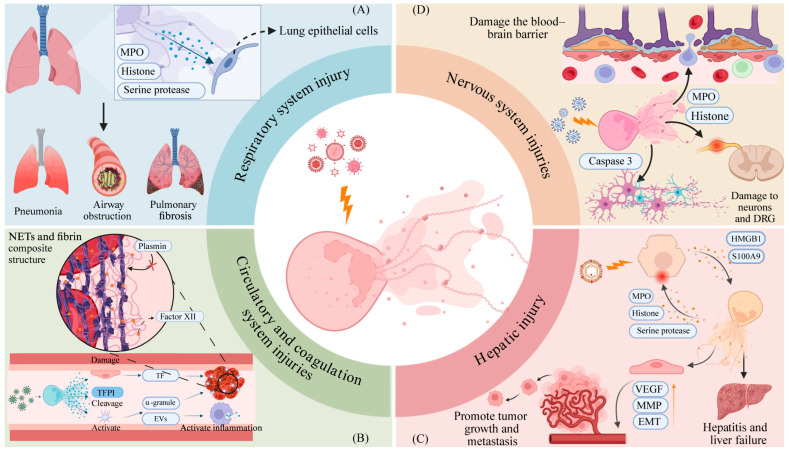
NET-mediated host immunopathological mechanisms. (**A**): Neutrophil-derived proteins are highly toxic to lung epithelial cells and contribute to the progression of diseases such as viral pneumonia, airway obstruction, and pulmonary fibrosis. (**B**): Virus-induced NETs can contribute to procoagulant activity through multiple mechanisms. Neutrophil-derived cytotoxic proteins can injure endothelial cells, leading to the exposure of TF. NETs may also promote the degradation of TFPI or activate platelets to release extracellular vesicles and α-granules enriched in TF, thereby enhancing coagulation and amplifying the immune response. Meanwhile, the negatively charged DNA backbone of NETs can attract the aggregation of coagulation factor XII, initiating the intrinsic coagulation pathway. Additionally, NETs and fibrin nets can form dense structures that resist the action of plasmin. (**C**): HBV stimulates hepatocytes to release HMGB1 and S100A9, which are DAMP molecules that activate NETosis. On the one hand, neutrophils further damage hepatocytes by releasing neutrophil-derived proteins, thereby exacerbating hepatitis and promoting liver failure. On the other hand, neutrophil-derived proteins promote the expression of VEGF and MMPs in endothelial cells and facilitate EMT, thereby promoting tumor angiogenesis and enhancing tumor growth and migration rates. (**D**): Virus-induced NETs disrupt the blood–brain barrier through neutrophil-derived proteins, damage DRGs and neurons, and induce neuronal apoptosis by activating caspase-3.

Neutrophils play a dual role in the initiation and progression of inflammation [74,75], and NETs, as toxic substances directly released into the internal milieu, exhibit similar dual effects (Figure 2). When the generation or clearance of NETs becomes imbalanced relative to viral infection levels, it may trigger intense immune responses. The key components of NETs, particularly histones, MPO, and neutrophil elastase (NE), possess broad cytotoxic properties and can directly interact with various cell types. This not only leads to direct tissue cell damage but also is closely associated with acute exacerbations and chronic sequelae of multiple viral infectious diseases [47,76,77]. Therefore, a thorough understanding of the mechanisms underlying NET-mediated immunopathology is of great significance for assessing disease severity, developing targeted intervention strategies, and improving patient outcomes.

### 6.1. SARS-CoV-2

In severe pneumonia caused by SARS-CoV-2, NETs are frequently overexpressed, and their components exhibit potent toxicity toward pulmonary epithelial cells [78]. This toxicity exacerbates patient conditions through a NET-IL-1β positive feedback loop, inducing and exacerbating multiple complications including acute lung injury (ALI), acute respiratory distress syndrome (ARDS), cytokine storm (CS), and macrophage activation syndrome (MAS) [76,77,78,79]. Beyond acute lung injury, persistent NET stimulation in chronic COVID-19 patients also induces alveolar epithelial–mesenchymal transition (EMT), pulmonary fibrosis, vascular inflammation, thrombosis, and myocardial fibrosis [80]. Given NETs’ pivotal role in COVID-19 lung pathology, their biomarkers have been employed to assess pneumonia severity at admission and predict patient prognosis [23,81].

The impact of SARS-CoV-2-induced NETs extends beyond pulmonary tissue, imposing a significant burden on the circulatory system. Their disruption of the coagulation system involves multifaceted synergistic effects. First, during pulmonary inflammation, recruited neutrophils form complexes with platelets in the lung, promoting platelet activation and the release of multiple coagulation factors [82]. Furthermore, the negatively charged DNA scaffold of NETs attracts coagulation factor XII, activating the intrinsic coagulation pathway [83]. Simultaneously, histones disrupt vascular endothelial integrity, exposing TF to activate prothrombin via the extrinsic pathway [84,85,86,87]. These dual pathways collectively accelerate the conversion of fibrinogen to fibrin. Furthermore, NE and cathepsin G (CG) can specifically degrade tissue factor pathway inhibitor (TFPI), disrupting the body’s regulatory mechanisms for anticoagulation [88]. Histones H3 and H4 also bind to multiple platelet surface receptors, further promoting platelet aggregation and α-granule release [89]. Last but not least, NETs, as fibrous network structures extending into the internal environment, can serve as platforms for the coagulation cascade. Through multiple mechanisms, they promote fibrin deposition and activation, trigger microthrombus formation, and accelerate DIC progression [44]. Electron microscopy reveals that NETs form dense complexes with fibrin, with the pore size of NETs’ meshwork (15–17 nm) being significantly smaller than that of isolated fibrin (100–200 nm) [6,90]. The intertwining of these two structures severely impedes the penetration of fibrinolytic enzymes, reducing thrombus degradation efficiency and further exacerbating microvascular obstruction. Concurrently, histones bind to fibrinogen and fibrin, further enhancing the structural stability of the NET–fibrin network and disrupting interactions between thrombin and coagulation regulatory proteins [91]. While this mechanism strengthens the stability of the NET–fibrin network and its ability to capture pathogens, it also increases the risk of NET-associated thrombotic diseases in the context of viral infection [92]. Autopsy studies reveal the extensive co-deposition of NETs and fibrin in the lung tissues of critically ill COVID-19 patients. CT imaging and histopathology confirm diffuse alveolar damage and hyaline membrane formation in the lungs, with extensive neutrophil infiltration and early fibrin thrombus formation visible within alveolar spaces and microvessels. The distribution of microthrombi highly overlaps with sites of alveolar hemorrhage [48]. Clinical data similarly indicate that NET-related biomarkers in COVID-19 patient plasma correlate closely with circulatory dysfunction. ICU patients exhibit significantly higher MPO-DNA levels than non-ICU patients [79], and NET-related markers such as H3Cit-DNA, cfDNA, and NE show significant positive correlations with D-dimer and plasmin-plasmin inhibitor complex levels [93]. Animal model studies further indicate that recombinant DNase I normalizes clotting times in mice with virus-induced respiratory failure, improves local tissue perfusion, and reduces mortality [94]. Collectively, these findings provide a comprehensive evidence chain for NET–circulatory system interactions, suggesting that the NET–coagulation axis may serve as a critical precision therapy target for COVID-19.

Beyond the respiratory and circulatory systems, the chronic inflammation and microvascular damage induced by sustained NET release over time can compromise the blood–brain barrier. This disruption allows immune cells and inflammatory cytokines to infiltrate neural tissue, ultimately leading to neuroinflammation and cognitive dysfunction in patients [80].

Therefore, NETs not only serve as mediators of immune-mediated tissue injury but also act as amplifiers of the inflammatory cascade. They link multiple pathological processes and play a pivotal role in both the acute injury and chronic sequelae of COVID-19.

### 6.2. Respiratory Syncytial Virus

Unlike SARS-CoV-2, RSV primarily affects children, causing lower respiratory tract infections (LRTIs) and airway obstruction [95,96]. Studies indicate that infected patients exhibit substantial NETs in pulmonary mucus plugs. In a bovine respiratory syncytial virus (bRSV)-induced LRTI calf model, the local administration of α-streptolysin significantly reduced airway NET levels, thereby alleviating obstruction, confirming the pathogenic role of NETs in RSV-induced airway obstruction [97,98]. Nevertheless, it should be noted that while NETs exhibit antiviral effects against RSV as described earlier, high concentrations of NETs (≥16 μg/mL) exert a dual effect: reducing viral load while enhancing cytotoxicity, synergistically exacerbating lung tissue damage [54,55]. Conversely, the combined administration of ibuprofen and fusion protein inhibitors suppresses NET formation by inhibiting the cyclooxygenase pathway, thereby reducing neutrophil activation and inflammatory cytokine release, thereby alleviating the symptoms of calf bRSV infection [99].

### 6.3. Influenza A Virus

IAV is a common respiratory pathogen that causes seasonal epidemics and occasional pandemics. Its subtypes, such as H1N1 and H3N2, exhibit significant morbidity and mortality rates globally. The virus primarily invades the respiratory tract, inducing acute inflammatory responses, with a higher likelihood of progressing to severe pneumonia, particularly in susceptible populations like children and the elderly [100]. Studies indicate that plasma and bronchoalveolar lavage fluid (BALF) from IAV pneumonia patients exhibit markedly elevated NET levels, with BALF NET concentrations exceeding plasma levels by more than tenfold. Similarly to SARS-CoV-2 infection, NETs contribute to IAV-induced inflammatory damage by impairing alveolar epithelial cells and lung tissue. However, it is important to note that only elevated plasma NETs correlate with poor prognosis in IAV patients. cfDNA-DNA and DNA–histone complexes can distinguish between mild and severe infections and predict influenza outcomes, providing a basis for early clinical intervention. In contrast, NETs in the alveoli have no prognostic value [101,102]. A possible explanation for this phenomenon is that the majority of severe H1N1 patients succumb to multiple organ dysfunction syndrome (MODS) rather than isolated respiratory failure. Alveolar NETs primarily reflect localized pulmonary inflammation and cannot effectively correlate with the severity of systemic multi-organ injury. In contrast, circulating NET levels better reflect systemic inflammatory responses and organ function, thereby exhibiting a stronger association with mortality and disease severity.

### 6.4. Other Respiratory Viruses

Asthma, a common chronic respiratory disease, is also closely related to the abnormal formation of NETs. RV not only induces asthma but also promotes neutrophil NETosis, exacerbating asthma symptoms through a positive feedback loop [40].

VZV infection in adults is often complicated by severe pneumonia, which is difficult to treat and has a high mortality rate [103]. Significantly elevated levels of NETs were detected in the alveolar lavage fluid and blood of varicella pneumonia patients, and their levels were positively correlated with disease severity [39]. Researchers have used a macaque model infected with simian varicella virus (SVV) to study the pathogenesis of varicella pneumonia. They found that following SVV infection in macaques, the virus replicates in alveolar epithelial cells and bronchial-associated lymphoid tissue and induces a significant early type I interferon response, recruiting neutrophils to the infected site. The neutrophils release NETs and other proteases that directly damage alveolar epithelial tight junction proteins and endothelial adherens junction proteins, exacerbating alveolar barrier damage [39].

### 6.5. Dengue Virus

In DV patients, excessive NETs also drive the pathological progression of dengue hemorrhagic fever through several mechanisms. On the one hand, neutrophil-derived proteins severely damage vascular endothelial cells, disrupting vascular endothelial tight junction proteins and leading to the loss of vascular endothelial integrity, ultimately resulting in vascular dysfunction [46,104]. On the other hand, DV can directly activate platelets, promoting the release of TF-rich extracellular vesicles. These vesicles activate macrophages to secrete large amounts of inflammatory cytokines, thereby facilitating the onset of dengue hemorrhagic fever through an immune cascade reaction [45]. Similarly to COVID-19 patients, plasma coagulation mediators such as D-dimer levels are significantly disrupted in severe DV patients [105]. However, no studies have yet reported the association between NET biomarkers and coagulation mediators in dengue patients, so the direct relationship between the two remains to be elucidated.

### 6.6. Coxsackievirus Group B

Coxsackievirus belongs to the genus Enterovirus of the family Picornaviridae. Among these, Coxsackievirus group B (CVB) exhibits cardiotropism and is the primary cause of viral myocarditis, accounting for 30–50% of cases [106]. In mice with viral myocarditis induced by CVB3, NETs were extensively deposited in the myocardial interstitium, highly overlapping with the areas of myocardial necrosis. At this stage, the myocardial fiber structure showed obvious fragmentation and edema, while NE in NETs was localized in areas of myocardial fibrosis and could disrupt the integrity of the myocardial basement membrane by degrading type IV collagen and laminin. Furthermore, studies have shown that early neutrophil depletion and PAD4 knockout can reduce myocardial inflammatory responses and decrease myocardial necrosis in mice [107].

### 6.7. Hepatitis B Virus

Hepatitis B is a liver disease caused by infection with HBV, which is characterized pathologically by hepatocellular injury, chronic inflammatory responses, and progressively worsening liver fibrosis [108]. Studies have shown that NET markers such as plasma MPO-DNA levels can serve as prognostic markers for predicting HBV-related liver failure and assist in clinical drug selection for patients with HBV-related liver failure [109]. The HBV stimulation of hepatocytes leads to the release of DAMPs such as HMGB1 and S100A9, which are inflammatory mediators that serve as key factors promoting the formation of pathogenic NETs, thereby exacerbating liver injury [34]. In a mouse model of fulminant viral hepatitis, the survival rate of mice increased from 4% to 29% after the DNase I degradation of NETs, with a significant improvement in liver function [35]. Additionally, HBV infection is a major risk factor for hepatocellular carcinoma, with most liver cancer patients having a history of HBV infection [110]. Studies have shown that the serum levels of MPO-DNA are significantly elevated in HBV-positive liver cancer patients, and co-culturing liver cancer cells with NETs in vitro stimulates lumen formation in HUVEC, promotes the expression of vascular endothelial growth factor (VEGF) and matrix metalloproteinases (MMPs), and facilitates EMT. More importantly, these mechanisms significantly enhance the proliferation, migration, and invasive capacity of hepatocellular carcinoma cells. Therefore, circulating NETs hold potential as alternative biomarkers for predicting HBV-associated hepatocellular carcinoma metastasis outside the liver [34]. This strongly suggests that NETs play a critical role in promoting angiogenesis and progression within the tumor microenvironment.

### 6.8. Zika Virus

ZIKV infection has been associated with various neurological disorders, including Guillain–Barré syndrome (GBS). In ZIKV-induced neurological damage, the NETs induced by ZIKV exhibit significant toxic effects on peripheral nerve cells. These effects include damage to dorsal root ganglion explants and associated cells, the inhibition of axonal growth, reduced cell viability, and the impairment of myelinating cells. Histones and MPO are considered key components responsible for DRG cell damage. Purified NETs and MPO significantly reduce synapse formation in neurons and also activate caspase-3, thereby inducing neuronal apoptosis.

## 7. Conclusions

The role of neutrophils in combating viral infections has gradually been elucidated, yet the underlying mechanisms remain complex with numerous gaps. Neutrophils form innate immune defenses by releasing NETs, but their uncontrolled release has become a key driver of multi-organ injury. During the early stages of viral infection, if neutrophils lack the capacity to respond to stimuli and actively release NETs, this may allow viruses to easily breach the innate immune barrier. Enhancing neutrophil activity to promote local NET release can strengthen antiviral immunity. However, during severe infection phases, imbalanced NET release may exacerbate cytokine storms and cellular tissue damage. Blocking excessive NET formation may require PAD4 enzyme inhibitors (e.g., GSK484) or ROS scavengers (e.g., N-acetylcysteine), while degrading released NETs could be achieved using DNases (e.g., DNase I and α-Streptodornase) to mitigate associated complications. In therapeutic studies for acute respiratory distress syndrome (ARDS) patients, recombinant DNase I effectively reduced inflammatory responses and coagulation abnormalities while improving lung structure and function. Research on traumatic brain injury (TBI) demonstrated that DNase I intervention improved neurological function scores and motor coordination by reducing NET deposition. However, it is noteworthy that DNase I only disrupts the DNA scaffold structure of NETs without effectively removing the various cytotoxic proteins attached to their surfaces. This limitation may constrain its efficacy in reducing tissue damage, mitigating inflammatory cell infiltration, and suppressing cytokine release. Future research may need to explore combination therapies with other protease inhibitors to address this limitation and enhance its therapeutic potential. Furthermore, DNase I usage requires precise dose optimization, as its application reduces NETs’ antiviral killing activity.

Clinically, NET-associated biomarkers demonstrate strong potential in assessing disease severity and prognosis in viral infectious diseases. In COVID-19, the serial monitoring of plasma MPO-DNA and CitH3 levels aids in identifying high-risk patients progressing to ARDS or thrombosis. In HBV-associated liver disease, combining MPO-DNA with routine liver function tests improves the accuracy of predicting 90-day mortality in ACLF patients, providing a basis for liver support therapy or transplantation evaluation. In pediatric RSV infections, detecting NET components in nasal aspirates can help in assessing airway obstruction severity, guiding drug use to reduce mucus blockage. Furthermore, the heterogeneity of NET components offers new directions for the stratified and personalized treatment of viral infectious diseases. For instance, dynamic changes in NET biomarkers may guide the timing of clinical interventions in patients with extrahepatic metastases of HBV-associated hepatocellular carcinoma.

However, current research remains limited. Most therapeutic strategies are still in the preclinical stage, clinical trial data are scarce, and the long-term safety of NET-targeted therapies has not been fully evaluated, all of which complicate clinical translation. Therefore, further investigation into the formation mechanisms and regulatory networks of NETs in the context of viral infection is crucial for harnessing this double-edged sword and refining personalized treatment strategies.

## Abbreviations

The following abbreviations are used in this manuscript:

ACLF	Acute-on-chronic liver failure
ALI	Acute lung injury
ARDS	Acute respiratory distress syndrome
B19V	arvovirus B19
BALF	Bronchoalveolar lavage fluid
BPI	Bactericidal/permeability-increasing protein
CG	Cathepsin G
CGD	Chronic granulomatous disease
CHIKV	Chikungunya virus
Cit H3	Citrullinated histone 3
CS	Cytokine storm
CVB	Coxsackievirus group B
DENV2	Dengue virus serotype 2
DV	Dengue virus
EBV	Epstein–Barr virus
EMT	Epithelial–mesenchymal transition
Env	Envelope glycoprotein
EXOs	Exosomes
FGL2	Fibrinogen-like protein 2
FVH	Fulminant viral hepatitis
GBS	Guillain–Barré syndrome
HBV	Hepatitis B virus
HCMV	Human cytomegalovirus
HIV	Human immunodeficiency virus
HMGB1	High mobility group box 1
HSV	Herpes simplex virus
HTNV	Hantavirus
IAV	Influenza A virus
KSHV	Kaposi’s sarcoma-associated herpesvirus
LRTIs	Lower respiratory tract infections
MAS	Macrophage activation syndrome
MCOLN3	Mucolipoprotein 3
MMPs	Matrix metalloproteinases
MODS	Multiple organ dysfunction syndrome
MPO	Myeloperoxidase
MVs	Microvesicles
N protein	Nucleocapsid protein
NE	Neutrophil elastase
NETs	Neutrophil extracellular traps
NS1	Non-structural protein 1
PAD4	Peptidyl arginine deiminase 4
pDCs	Chronic granulomatous disease
PE	Preeclampsia
PRRs	Pattern recognition receptors
PSGL-1	P-selectin glycoprotein ligand 1
ROS	Reactive oxygen species
RSV	Respiratory syncytial virus
RV	Rhinovirus
S protein	Spike protein
SARS-CoV-2	Severe acute respiratory syndrome coronavirus 2
STBMs	Syncytiotrophoblast-derived microparticles
SVV	Simian varicella virus
TF	Tissue factor
TFPI	Tissue factor pathway inhibitor
VEGF	Vascular endothelial growth factor
VWF	von Willebrand factor
VZV	Varicella-zoster virus
ZBP1	Z-DNA-binding protein 1
ZIKV	Zika virus
